# Icing the Pain–Ultrasound-Guided Cryoablation of Symptomatic Post-Amputation Stump Neuroma

**DOI:** 10.1007/s00270-021-02998-9

**Published:** 2021-11-24

**Authors:** C. von Falck, M. Orgel, F. Wacker, H. H. Aschoff, C. Krettek, K. I. Ringe

**Affiliations:** 1 Department of Diagnostic and Interventional Radiology, Hannover Medical School, Carl-Neuberg Str. 1, 30625 Hannover, Germany; 2 Department of Trauma Surgery, Hannover Medical School, Carl-Neuberg Str. 1, 30625 Hannover, Germany

**Keywords:** Cryoablation, Ultrasound, Stump neuroma, Amputation, Pain

## Abstract

**Purpose:**

To assess the feasibility and outcome of ultrasound-guided cryoablation in patients with a sensitized stump neuroma after limb amputation.

**Material and Methods:**

Seven patients (3 females, 4 males; mean age 42 years) were included in this retrospective study. Ultrasound-guided cryoablation of a previously identified painful stump neuroma was performed. Pain was assessed on the visual analogue scale (VAS) and compared before and after cryoablation (Wilcoxon Test). The degree of pain alleviation was correlated with patient age, duration of pain before ablation and time interval between amputation and ablation (Spearman correlation). A *p*-value less than 0.05 was deemed statistically significant.

**Results:**

Nine cryoablations were performed for 8 neuromas. Technical success was 100%, there were no major complications. Mean follow-up was 27 months. There was a statistically significant decrease of pain from mean 8.3 / 10 (baseline) to 4 /10 (on day one), 2.1 / 10 (at one week) and 3 / 10 (at last follow-up) (*p* < 0.05). Patient satisfaction with cryoablation treatment was very high (median score 70 / 100).

**Conclusion:**

In our small population observational study, ultrasound-guided cryoablation of a sensitized stump neuroma was effective and safe for pain alleviation with very good long-term results.

**Level of Evidence:**

Level 2, Observational study with dramatic effect

## Introduction

Approximately 50.000–70.000 amputations are performed each year in Germany [1]. In 2005, about 1.6 million people in the US were living with limb loss and studies estimate that this number will double by 2050 [2]. Up to 85% of amputees complain of post-amputation pain, typically perceived as stump and / or phantom limb pain [3, 4]. Particularly, in case of pain in the stump, a sensitized neuroma may be identified in up to 48.7% as a cause [4]. However, there is no standard therapy for post-amputation pain and up to date especially those affected often perceive overall treatment as unsatisfactory.

Experience with cryoablation for treatment of neuroma is very limited and mostly derived from treatment of Morton Neuroma [5, 6]. Based on the mechanism of tissue, i.e., nerve destruction and the known intrinsic analgesic effect, the application of cryoablation for treatment of a sensitized stump neuroma after limb amputation seems obvious. The purpose of our study was therefore to prospectively evaluate the long-term outcome of cryoablation in patients with post-amputation pain, in whom a sensitized stump neuroma had been identified.

## Materials and Methods

### Patients

This retrospective observational study of prospectively collected data was IRB-approved. Informed consent was obtained from all patients. Patients were referred for cryoablation of a symptomatic stump neuroma between March 2018 and December 2020. Inclusion criteria were as follows: patient age ≥ 18 years, adequate coagulation status, on imaging recognizable painful stump neuroma and decrease of pain after probatory perineural infiltration. The final study population comprised seven patients (3 females, 4 males; mean age 42 years; Table 1). Prior to ablation, all patients complained of typical neuropathic pain in the stump, restricting them in their daily living activities, Additional complaints were local cramps, phantom pain and an inability or limitation in wearing their limb prosthesis.

**Table 1:** Demographic and clinical data of seven patients in whom ultrasound-guided cryoablation of a painful stump neuroma was performed

	All cryoablations
Total number of patients	7
Total number of neuromas	8
Total number of cryoablations	9*
Sex [number of males / females]	4/3
Age [years]	42 (25–55)
Site of amputation [upper leg / lower leg]	2/5
Type of rehabilitation [shaft prosthesis / endo-exo-prostheses]	4/3

If not stated otherwise, data are presented as mean with the range in parentheses.

*Includes one patient, in whom a second cryoablation was performed for the same neuroma during follow-up due to aggravating pain after initial pain alleviation, and another patient, in whom two different neuromas were treated consecutively

### Intervention

Ultrasound-guided interventions in patients with symptomatic stump neuroma were performed in two steps, by two board-certified radiologists with more than 10 years of clinical experience in percutaneous image-guided procedures. First, clinical and sonographic evaluation of the stump was performed. If a neuroma was identifiable, image-guided perineural infiltration with 5 ml prilocaine and 5 ml ropivacaine was performed using a 20G needle. Patients were monitored for two hours after the injection and discharged with the request to document their pain over the following 7 days.

If pain decreased after perineural infiltration as compared to pre-infiltration pain, cryoablation was performed as an inpatient procedure under analgosedation and ultrasound guidance (Visual Ice System™; Galil Medical Inc., Arden Hills, USA). A single cryoprobe (IceSphere™ or IceSeed™) was advanced to the previously identified neuroma and ablation started (2 cycles of 6 min freezing separated by a 4-min thaw cycle). The ablation zone and surrounding structures were continuously monitored with ultrasound. Care was taken that the resulting ice ball covered the whole neuroma without extending less than 0,5 cm to the skin surface. In addition, skin protection using warm gel cushions was performed. Patients were discharged the next morning, after follow-up ultrasound. Technical success of the procedure (i.e., that the neuroma was treated according to the protocol and covered completely by the ablation zone) [7] and complications [8] were assessed.

### Pain Assessment and Follow-up

Pain intensity before and after cryoablation was evaluated using the visual analogue scale (VAS; 0–10). Outpatient clinical follow-up was scheduled 6 weeks after the ablation. At final follow-up, patients were asked how satisfied they were with the cryoablation treatment (scale 0–100; 0 = not satisfied at all; 100 = absolutely satisfied) and whether they would be willing to undergo this treatment again, if needed.

### Statistical Analysis

Statistical analysis was performed using GraphPad Prim (version 7; GraphPad Software, Inc., USA). Pain scores before and after cryoablation were compared (Wilcoxon-Test). The degree of pain alleviation after cryoablation was correlated with patient age, duration of pain before ablation and time interval between amputation and ablation (Spearman correlation; *p*-value < 0.05 deemed statistically significant).

## Results

### Patients and Interventions

**Fig. 1:**
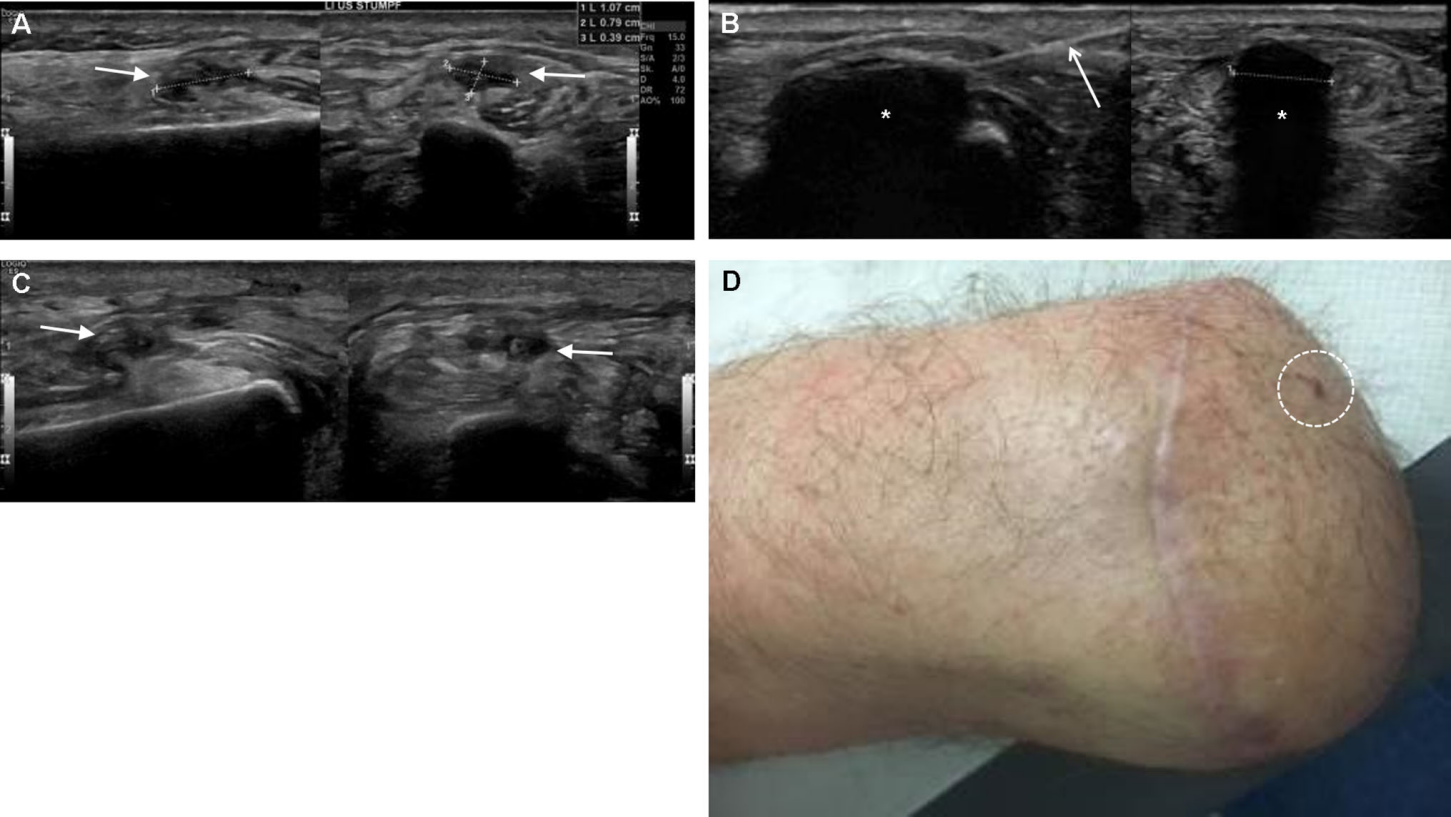
Exemplary cryoablation of a painful stump neuroma in a 33-year-old male patient, 18 months post traumatic lower limb amputation. The patient suffered from massive neuropathic pain (10 / 10 on the VAS) including hyperpathia despite pain medication, and was thus not able to wear his prosthesis. (Ultrasound images of the neuroma (arrows in A-C) are provided in the longitudinal (left) and transversal (right) plane, respectively. **A** Depiction of a typical neuroma (11 × 8x4mm) causing pain, which could be triggered by pressure. **B** Ultrasound-guided placement of the cryoprobe (open arrow) and monitoring of the evolving ice ball (*) that covers the neuroma completely. **C** Image control one day following the intervention. Discrete residual soft tissue edema surrounding the neuroma can be appreciated. **D** Clinical impression of the stump one day after cryoablation, only the site of probe insertion (circle) is visible. At this time, pain had declined to 2 / 10 on the VAS and the patient was able to wear his limb prosthesis again)

A total of nine cryoablation procedures for treatment of painful stump neuroma were performed in seven patients (Fig. 1, Table 1). Overall technical success of cryoablation was 100%. In one patient, local redness of the skin was visible one day after ablation, which resolved spontaneously without any specific treatment (grade 1 complication).

### Pain Course and Follow-up

**Fig. 2:**
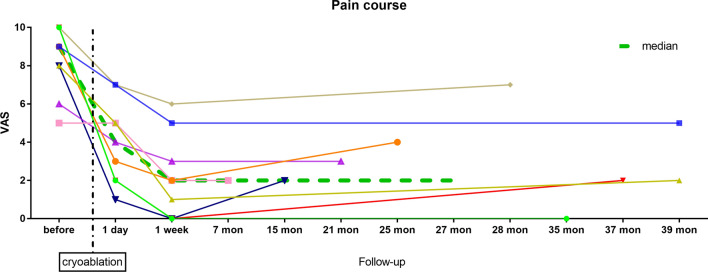
Pain assessment before and after cryoablation of painful stump neuroma in individual patients. (*VAS*, Visual analogue scale)

**Fig. 3:**
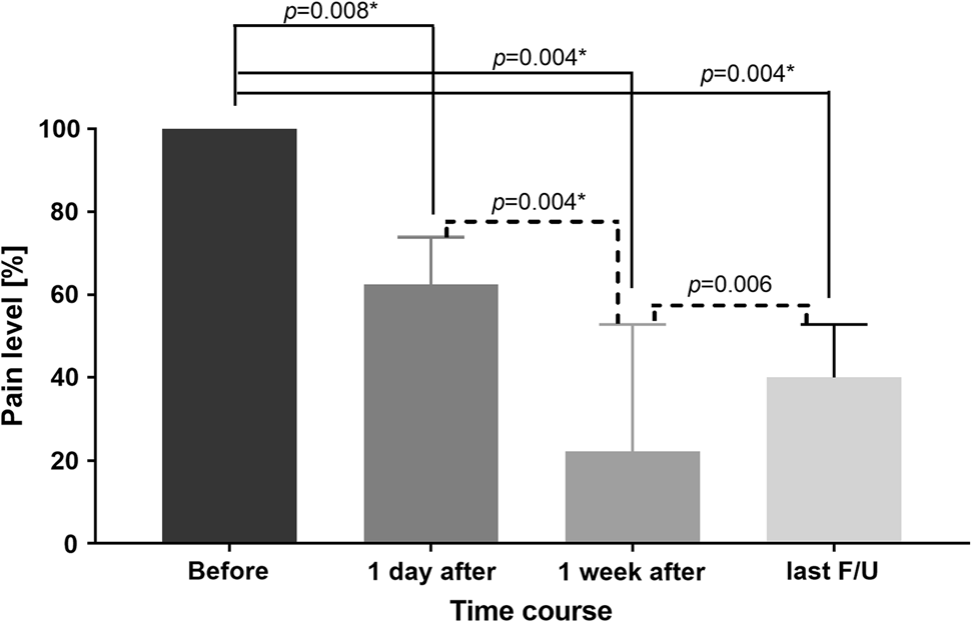
Comparison of pain level before and after cryoablation for painful stump neuroma. (*F/U*, last follow-up; *indicates a statistically significant difference)

Mean follow-up after cryoablation was 27 months (range 6.8–40 months). Mean pain score on the VAS before cryoablation was 8.3/10 (range 5–10). All patients reported marked and statistically significant pain alleviation after cryoablation (Table 2, Fig. 2, 3). The lowest pain level after cryoablation (NADIR) was observed in all patients 1 week after ablation (mean score 2.1/10, range 0–6). There was no correlation of pain decrease with patient age, duration of pain before ablation or time interval between amputation and ablation (*p* > 0.05). Patient satisfaction with cryoablation treatment was very high (median score 70/100).

**Table 2:** Results of ultrasound-guided cryoablations in seven patients, including one patient with re-ablation of a single neuroma and one patient with two neuromas that were treated consecutively

	Intervention-based analysis
Total number of cryoablations	*n* = 9*
Time interval between amputation and cryoablation [months]	100 (11–336)
Duration of pain allegedly caused by neuroma [months]	8.4 (2–24)
Pre-interventional pain intensity^#^	8.3 (5–10)
Post-interventional pain intensity^#^	
1 day	4 (1–7)
1 week	2.1 (0–6)
at last follow-up	3 (0–7)
Follow-up after cryoablation [months]	27 (6.8–40)
Satisfaction with intervention^§^	69 (10–100)
Willingness to undergo re-intervention [yes / no]	6 / 1^#^

Unless stated otherwise, data are presented as mean with the range in parentheses.

^#^Assessed on the visual analogue scale (VAS); ^§^on a scale from 0 to 100.

*Includes one patient, in whom a second cryoablation was performed for the same neuroma during follow-up due to aggravating pain after initial pain alleviation, and another patient, in whom two different neuromas were treated consecutively.

^#^The single patient that was not satisfied with treatment outcome (satisfaction score 10 / 100) had a 30% reduction of pain after cryoablation and indicated that he would not be willing to undergo re-ablation

## Discussion

In the present study, it was demonstrated that ultrasound-guided cryoablation of a previously identified sensitized stump neuroma may lead to a significant and persistent decrease of pain. Interventions were safe, and patient satisfaction with this treatment was very high. Potential advantages over for example radiofrequency ablation or alcohol injection include the known intrinsic analgesic effect of cryoablation along with preservation of the primary nerve structure (Wallerian degeneration) [9], and better controllability of the ablation zone.

To the best of our knowledge, there are only two studies so far in which cryoablation for a painful neuroma was performed specifically in the setting of post-amputation pain. The results of our present study are comparable to those two published case series, but of note, follow-up in our study was distinctly longer. Neumann et al. performed cryoablation as an alternative to phenol injections in 10 patients with a neuroma and therapy refractory stump pain [10]. Even though the degree of pain decrease was not further quantified, at 3 months follow-up 9/10 patients reported that pain had improved, and at 1 year follow-up 7/10 patients had returned to pretreatment levels of pain. The treatment protocol was slightly different with shorter freeze–thaw cycles (60 s). Furthermore, the cryoprobes used were quite small (2 mm) as compared to those used in our study. It remains unclear, whether the neuroma was covered completely by the ice ball in each patient, and incomplete ablation could be an explanation why the majority of patients experienced the same pain one year after treatment as compared to the baseline level. In the largest study to date, Prologo et al. performed cryoablation in 21 amputees, including 13 patients with a sensitized stump neuroma [11]. At one week follow-up, mean pain decrease was 13% (compared to 73% in our study). At last follow-up, which occurred at a mean of approximately 6.4 months, pain decrease was 67.7%, as compared to 63% in our study at a mean follow-up of 27 months.

This study has some limitations. First, the number of patients included is small. Secondly, even though this was a prospective study, patients’ quality of life before and after treatment was not assessed in detail, e.g., with a standardized questionnaire or tools dedicated for assessment of neuropathic pain.

In conclusion, the results our prospective small cohort observational study show that ultrasound-guided cryoablation of a sensitized stump neuroma in patients with pain may be a safe and effective treatment with very encouraging short- and long-term results.
